# Which subgroup of patients with rheumatoid arthritis benefits from switching to rituximab versus alternative anti-tumour necrosis factor (TNF) agents after previous failure of an anti-TNF agent?

**DOI:** 10.1136/ard.2008.105064

**Published:** 2009-05-15

**Authors:** A Finckh, A Ciurea, L Brulhart, B Möller, U A Walker, D Courvoisier, D Kyburz, J Dudler, C Gabay, on the behalf of the doctors of the Swiss Clinical Quality Management Programme for Rheumatoid Arthritis

**Affiliations:** 1 Division of Rheumatology, University Hospital of Geneva, Geneva, Switzerland; 2 Department of Rheumatology, University Hospital of Zurich, Zurich, Switzerland; 3 Department of Rheumatology, Clinical Immunology and Allergy, University Hospital of Bern, Bern, Switzerland; 4 Division of Rheumatology, University Hospital of Basel, Basel, Switzerland; 5 Division of Clinical Epidemiology, University Hospital of Geneva, Geneva, Switzerland; 6 Division of Rheumatology, University Hospital of Lausanne, Lausanne, Switzerland

## Abstract

**Background:**

Patients with rheumatoid arthritis (RA) with an inadequate response to TNF antagonists (aTNFs) may switch to an alternative aTNF or start treatment from a different class of drugs, such as rituximab (RTX). It remains unclear in which clinical settings these therapeutic strategies offer most benefit.

**Objective:**

To analyse the effectiveness of RTX versus alternative aTNFs on RA disease activity in different subgroups of patients.

**Methods:**

A prospective cohort study of patients with RA who discontinued at least one aTNF and subsequently received either RTX or an alternative aTNF, nested within the Swiss RA registry (SCQM-RA) was carried out. The primary outcome, longitudinal improvement in 28-joint count Disease Activity Score (DAS28), was analysed using multivariate regression models for longitudinal data and adjusted for potential confounders.

**Results:**

Of the 318 patients with RA included; 155 received RTX and 163 received an alternative aTNF. The relative benefit of RTX varied with the type of prior aTNF failure: when the motive for switching was ineffectiveness to previous aTNFs, the longitudinal improvement in DAS28 was significantly better with RTX than with an alternative aTNF (p = 0.03; at 6 months, −1.34 (95% CI −1.54 to −1.15) vs −0.93 (95% CI −1.28 to −0.59), respectively). When the motive for switching was other causes, the longitudinal improvement in DAS28 was similar for RTX and alternative aTNFs (p = 0.40). These results were not significantly modified by the number of previous aTNF failures, the type of aTNF switches, or the presence of co-treatment with a disease-modifying antirheumatic drug.

**Conclusion:**

This observational study suggests that in patients with RA who have stopped a previous aTNF treatment because of ineffectiveness changing to RTX is more effective than switching to an alternative aTNF.

Tumour necrosis factor antagonists (aTNFs) are very effective at improving the symptoms and signs of rheumatoid arthritis (RA) and at preventing structural joint damage.^1^ ^2^ ^3^ ^4^ However, not all patients with RA respond to aTNFs and about one-third of all patients with RA fail to achieve even a modest improvement of 20% in American College of Rheumatology criteria in large randomised controlled trials (RCTs).^5^ Furthermore, some patients discontinue aTNF because of adverse events (AEs) or the development of a secondary resistance, with gradual loss of effectiveness of these agents.^6^

Until recently, therapeutic options were limited for patients not responding satisfactorily to an aTNF. Despite a similar mode of action within the aTNF class, switching from one aTNF to another was the established treatment approach for patients for whom an aTNF failed or who did not tolerate an initial aTNF.^7^ The rationale for switching between aTNFs resides in variations in the chemical structure, in pharmacokinetic properties, in the stability of the TNF inhibitor complex and in the incidence of drug-neutralising antibodies between these agents.^8^ In patients for whom etanercept produced an inadequate response, one small randomised trial suggested a more favourable response for patients who switched to infliximab compared with those maintaining treatment with etanercept.^9^ From observational studies, we know that the effectiveness of subsequent aTNFs differs according to the reasons for switching.^10^ ^11^ ^12^

Biological agents with a different mechanism of action have become available, such as interleukin (IL) 1 inhibitors, IL6 inhibitors, B-cell depleting antibodies, or inhibitors of T-cell co-stimulation. A rationale for introducing biological agents with a different mode of action after a previous aTNF failure may be to overcome an aTNF class effect, particularly in cases of primary failure or recurrence of class-associated AEs. Several of these alternative biological agents have proved to be effective in patients with a history of prior aTNF failure in large RCTs against placebo.^13^ ^14^ ^15^ However, head-to-head trials comparing pertinent therapeutic options are missing. Small observational studies suggested that rituximab (RTX) may be more effective at controlling disease activity than an alternative aTNF in a population of patients with RA with an inadequate response to one or more aTNF.^16^ ^17^ ^18^ ^19^ A previous analysis of approximately 100 patients with RA from the Swiss RA cohort observed a more favourable evolution of 28-joint count Disease Activity Scores (DAS28) in the group that received RTX compared with alternative aTNFs,^16^ but the reasons leading to treatment switches were not examined. Patients may interrupt aTNF therapy for various reasons and it remains unclear in which clinical setting each therapeutic strategy offers most benefit.

The aim of this study was to analyse the effectiveness of switching to an alternative aTNF compared with initiating RTX in different subgroups of patients. In particular, we studied the influence on RA disease activity of the reason for switching, the type of aTNF switch, the number of previous aTNF failures and the presence of concomitant disease-modifying antirheumatic drugs (DMARDs).

## Methods

### Study population

Swiss Clinical Quality Management in rheumatoid arthritis (SCQM-RA) is a Swiss RA cohort, which has been described in detail elsewhere (online supplementary appendix).^20^ ^21^ Inclusion criteria for this analysis were a diagnosis of RA by a rheumatologist, discontinuation of an aTNF (infliximab, etanercept or adalimumab) followed by the initiation of either a second or a third alternative aTNF or a first course of RTX. We further required a baseline assessment of the DAS28 at the time of the new treatment initiation and at least one follow-up assessment within the first 12 months. The only exclusion criterion was RTX treatment for lymphoma. We censored observations after aTNF treatment interruption or RTX re-treatment. The analysis includes data collected between January 1998 and the end of March 2008.

### Study design

This was a longitudinal cohort study of patients with RA for whom at least one previous aTNF had produced an inadequate response. The study’s predefined primary outcome was the longitudinal improvement of RA disease activity as measured by the DAS28, a validated tool for the assessment of disease activity in RA.^22^ We used the DAS28 based on three variables, including the erythrocyte sedimentation rate, the number of swollen joints and the number of tender joints.^22^ The DAS28 ranges from 0.1 to 9.2, where 9.2 represents maximum disease activity.

Because the aim of this study was to analyse the relative benefit of RTX compared with alternative aTNF in different subgroup of patients, we selected a priori four potential effect modifiers: the reason for switching, the type of aTNF switch, the number of previous aTNF failures, and the presence of co-treatment with conventional DMARDs (supplementary appendix).

### Analysis

Baseline disease and treatment characteristics were compared between the two groups using the Student *t* test for normally distributed variables, the Mann–Whitney U test for non-normally distributed variables and the Pearson χ^2^ test for dichotomous variables. Fewer than 5% of covariates were sporadically missing; in order to minimise potential bias, we used the population average of the respective covariates as a substitute. All statistical tests were evaluated at the 0.05 significance level and were two-sided. Statistical analysis was performed with Stata version 9.2 (Stata Statistical Software, Texas, USA).

Since this is an observational study, selection bias is a concern because treatment assignment of RTX versus an alternative aTNF is not random. In fact, baseline characteristics suggest that for rheumatoid factor (RF)-positive patients and patients for whom an aTNF had been ineffective, treatment was preferentially started with RTX. Because such differences may substantially influence the evolution of disease activity, we used a propensity score approach to overcome such confounding effects and make the groups comparable for covariates believed to be associated with disease progression and to minimise the possibility of confounding by indication (supplementary appendix).^23^ The propensity score was used to stratify patients into five blocks with similar baseline characteristics. The evolution of disease activity outcomes was analysed using generalised mixed models for longitudinal data, adjusting for potential confounding factors using the propensity score stratas and the potential effect modifiers (supplementary appendix). We explored potential effect modification by the reason for switching, the type of aTNF switch, the number of previous aTNF failures and the presence of concomitant DMARDs using an interaction term.

## Results

Out of a total 479 patients in the SCQM-RA registry who received RTX or a second or third aTNF, 99 patients (21%) were excluded because of missing baseline DAS assessment (assessment not on the precise day of treatment switch), 59 patients (12%) were excluded because of missing follow-up assessments within the first year and three patients (1%) were excluded because RTX was administrated for concomitant lymphoma and not primarily for the treatment of RA. Excluded subjects were similar in their socioeconomic and disease characteristics (data not shown).

A total of 318 patients with RA met study inclusion criteria; 155 patients received RTX and 163 patients received a second or third aTNF. The 318 patients had an average of 3.6 assessments during a median follow-up period of 11 months (interquartile range (IQR) 6–12). At baseline, there were some differences between the RTX and the alternative aTNF groups (table 1). Patients in the RTX group were more often RF positive, had higher baseline DAS28 levels, higher Health Assessment Questionnaire scores and had tried more aTNF agents that had failed. Differences in baseline disease activity were partially explained by dissimilar causes of previous aTNF failure: patients given RTX preferentially after aTNF inefficacy (82% versus 51%, p<0.001), and a history of aTNF inefficacy was significantly associated with higher baseline DAS levels (4.88 vs 3.83 for other motives of aTNF discontinuation, p<0.001). After propensity score stratification, all baseline predictors were balanced, without significant differences between groups. In particular, no difference in baseline DAS28 levels (p = 0.73) or in RF positivity (p = 0.97) remained.

**Table 1:** Baseline patient and treatment characteristics

Disease characteristics	RTX (n = 155)	Alternative aTNF (n = 163)	p Value
Age (years), mean (95% CI)	55 (53 to 57)	55 (53 to 57)	0.70
Gender, male (%)	23	22	0.77
Educational level (years), median (IQR)*	12 (9–12)	12 (9–12)	0.18
RF (%)	88	77	0.02
Disease duration (years), mean (95% CI)†	11.9 (10.5 to 3.2)	10.7 (9.5 to 11.8)	0.18
Disease activity (DAS28), mean (95% CI)	4.99 (4.8 to 5.2)	4.08 (3.9 to 4.3)	<0.001
Functional disability (HAQ), median (IQR)‡	1.60 (1.10–2.00)	1.42 (0.96–1.85)	0.03
Concomitant DMARD use§			
Methotrexate (%)	67	61	0.20
Leflunomide (%)	17	19	0.72
Other DMARDs (%)	6	3	0.32
None (%)	18	19	0.83
Glucocorticoids (%)¶	58	55	0.71
Previous aTNF agents (n), median (IQR)	2 (1–2)	1 (1–1)	<0.001
Single previous aTNF agent (%)	43	88	<0.001
Two or more previous aTNF agents (%)	57	12	<0.001
Cause of previous aTNF interruption**			
Ineffectiveness (%)	82	51	<0.001
Adverse events (%)	24	33	0.09
Other (%)	3	18	<0.001

*Educational level, total number of years of school and college; †disease duration, disease duration at inclusion in years; ‡HAQ missing in 89 patients (28%); §The DMARD percentages represent the use of each co-therapy DMARD at baseline; patients could receive more than one DMARD co-therapy, which explains why the total may exceed 100%. ¶Glucocorticoids, concomitant low dose oral glucocorticoids; **Patients could discontinue aTNF owing to ineffectiveness (primary or secondary aTNF resistance), owing to adverse events or for other motives (patient preference, pregnancy wish, prolonged travel, etc). The total may exceed 100% because more then one cause could motivate aTNF interruption or different causes might have motivated aTNF discontinuations when patients had received more then one aTNF that had failed.

aTNF, tumour necrosis factor antagonist; DAS28, 28-joint count Disease Activity Score; DMARD, disease-modifying antirheumatic drug; HAQ, Health Assessment Questionnaire; RF, rheumatoid factor; RTX, rituximab.

Patients in the RTX group received a single course of RTX (two infusions of 1000 mg) with concomitant glucocorticoids, according to the manufacturer’s indications. In the alternative aTNF group, 51% of patients received adalimumab 40 mg subcutaneously every 2 weeks, 26% received etanercept 50 mg/week and 23% received infliximab intravenously, starting with 3 mg/kg.

**Figure 1:**
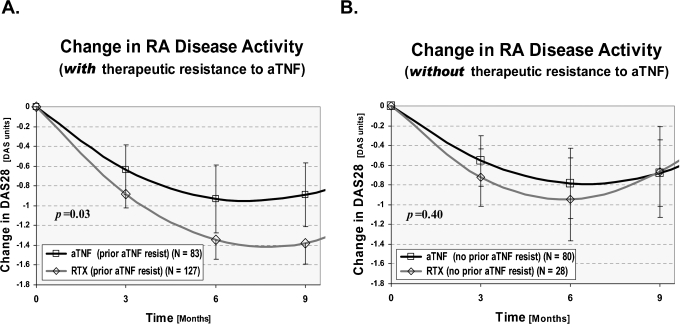
Effect modification by prior aTNF ineffectiveness. Change in rheumatoid arthritis (RA) disease activity after initiation of an alternative tumour necrosis factor antagonist (aTNF) or rituximab (RTX). The longitudinal improvement in RA disease activity (28-joint count Disease Activity Score (DAS28)) over the average treatment time is represented: (A) for patients switching because of ineffectiveness to the previous aTNF and (B) for patients switching because of adverse effects to the previous aTNF or other reasons. The improvement of DAS28 was more favourable with RTX only for patients with a history of prior aTNF ineffectiveness (effect modification  = 0.005). The progression trajectories depicted are adjusted for differences in baseline disease characteristics and treatment characteristics (supplementary appendix). Vertical lines represent the 95% confidence interval of the mean (only lower-bound interval for the RTX estimates).

The longitudinal improvement in DAS28 was overall more favourable in the RTX group than in the alternative aTNF group during the first year (p = 0.016). However, the relative benefit of RTX varied with the cause of prior aTNF failure (effect modification, p = 0.005; (supplementary appendix, table 2)). When the cause for switching was ineffectiveness to a previous aTNF, the longitudinal improvement in DAS28 was significantly better for RTX than for an alternative aTNF (p = 0.03; fig 1A). In this subgroup, at 6 months the decrease in DAS28 was −1.34 (95% CI −1.54 to −1.15) with RTX and −0.93 (95% CI −1.28 to −0.59) with alternative aTNFs. This represents 61% of patients with RTX compared with 37% with aTNF who had an improvement in DAS28 of more then 1.2 units, a clinically meaningful difference (p = 0.001).

When the motive for switching was not ineffectiveness (an AE or other reason), the longitudinal improvement in DAS28 was similar for RTX and for alternative aTNFs (p = 0.40; fig 1B). At 6 months the decrease in DAS28 was −0.94 (95% CI −1.37 to −0.52) with RTX and −0.78 (95% CI −1.14 to −0.43) with alternative aTNFs. The percentage of patients whose DAS28 score improved by more then 1.2 units at 6 months was also similar in the two groups (39% with RTX vs 28% with an aTNF, p = 0.28). Overall, the magnitude of DAS28 improvements was smaller in patients having switched treatment for reasons other than ineffectiveness, which is related to lower baseline DAS28 levels in this subgroup of patients. Patients experiencing an AE do not necessarily present with an RA flare at the time of treatment switch and therefore start the new treatment with lower baseline levels of disease activity, which results in smaller effect sizes.

**Figure 2:**
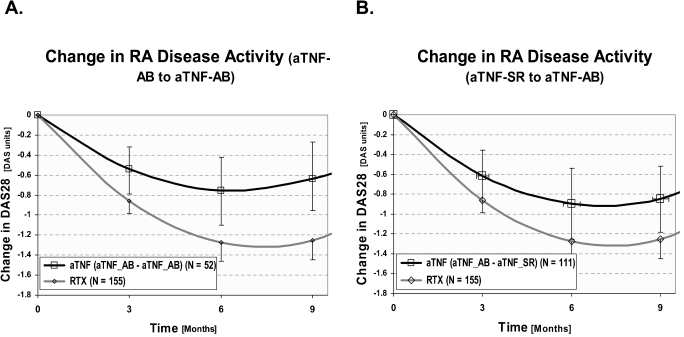
Effect modification by the type of aTNF switch. Change in rheumatoid arthritis (RA) disease activity after initiation of an alternative tumour necrosis factor antagonist (aTNF) versus rituximab (RTX). The longitudinal improvement in RA disease activity (28-joint count Disease Activity Score (DAS28)) over the average time on treatment is represented: (A) for patients switching from one aTNF monoclonal antibody (aTNF-AB) to another aTNF-AB; (B) for patients switching from an aTNF soluble receptor (aTNF-SR) to an aTNF-AB. No significant effect modification existed by the type of aTNF switch (p = 0.27). The progression trajectories depicted are adjusted for differences in baseline disease characteristics and treatment characteristics (supplementary appendix). Vertical lines represent the 95% confidence interval of the mean (only lower-bound interval for the RTX estimates).

When initiating an alternative aTNF, a majority of patients (68%) switched from an aTNF monoclonal antibody to a TNF soluble receptor or vice versa. No effect modification by the type of aTNF switch was found (p = 0.27; fig 2), The longitudinal improvement in DAS28 was overall more favourable with rituximab than with an alternative aTNF, irrespective of the type of TNF switch, despite a suggestion that patients switching from one anti-TNF monoclonal antibody to another anti-TNF monoclonal antibody had smaller responses than patients switching from an anti-TNF monoclonal antibody to a TNF soluble receptor (DAS28 improvement at 6 months −0.75 (95% CI −0.42 to −1.09) versus −0.90 (95% CI −0.61 to −1.18), respectively).

**Figure 3:**
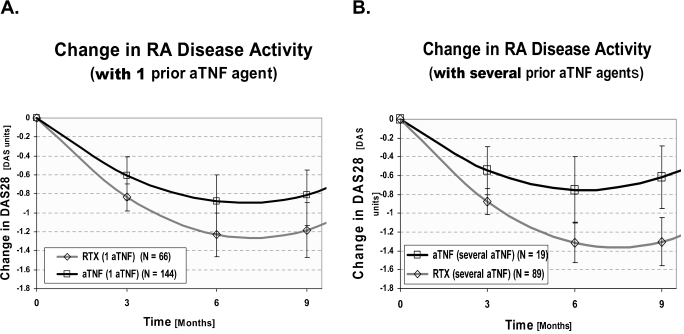
Effect modification by the number of prior aTNF failures. Change in rheumatoid arthritis (RA) disease activity after initiation of an alternative tumour necrosis factor antagonist (aTNF) versus rituximab (RTX). The longitudinal improvement in RA disease activity (28-joint count Disease Activity Score (DAS28) over the average time on treatment is represented: (A) for patients switching after a single prior aTNF failure and (B) for patients switching after multiple prior aTNF failures. No significant effect modification existed by the type of aTNF switch (p = 0.61). The progression trajectories depicted are adjusted for differences in baseline disease characteristics and treatment characteristics (supplementary appendix).

Furthermore, no effect modification appeared to exist according to the number of previous aTNF failures (p = 0.61; fig 3), indicating that the relative benefit of RTX was similar after one or several inadequate responses to aTNF. After one prior aTNF failure, the decrease in DAS28 at 6 months was −1.23 (95% CI −1.46 to −1.00) with RTX and −0.88 (95% CI −1.16 to −0.60) with alternative aTNFs, while after several aTNF failures, the decrease in DAS28 was −1.31(95% CI −1.53 to −1.10) with RTX and −0.75 (95% CI −1.18 to −0.32) with alternative aTNFs. We also could not demonstrate significant effect modification by concomitant DMARD use (p = 0.85) or concomitant methotrexate use (p = 0.27). Overall, in 8% of all patients, doctors reported an AE, with no significant difference between the two groups (p = 0.12). The number of events was too small to analyse differences in specific AEs between the groups.

## Discussion

This study confirms the results of small observational studies suggesting that biological agents of a different class, such as RTX, control RA disease activity more effectively than alternative aTNF agents in patients with an inadequate response to aTNF therapy.^16^ ^17^ ^18^ ^19^ However, the relative benefit of RTX varies with the reason for interrupting prior aTNFs (effect modification). When the motive for switching was ineffectiveness to an aTNF, the longitudinal improvement in RA disease activity was significantly more favourable with RTX than with an alternative aTNF agent. When the motive for switching was a cause other than ineffectiveness—namely, an AE or a personal preference, we found no advantage of RTX compared with a second or third aTNF. Overall, the magnitude of DAS28 improvements was slightly smaller than in a pivotal trial of RTX in this indication,^24^ which is certainly related to different study populations and lower disease activity levels at baseline.

With the growing availability of expensive therapeutic agents in RA, it becomes increasingly important to tailor the treatment to the individual patient in order to maximise the cost–benefit and to minimise the time for which suboptimal treatments are given. Patients for whom aTNF fail have different treatment options, including using a combination of conventional DMARDs, switching to an alternative aTNF, or changing to an agent with a different mechanism of action.^7^ ^15^ ^24^ Available evidence indicates that switching to a different aTNF agent may work^10^ ^25^; however, several cohort studies have demonstrated that effectiveness declines and drug retention decreases in patients who have received one or more TNF inhibitor previously.^10^ ^26^ ^27^ ^28^

The rationale for introducing agents with a different mode of action is to overcome problems related to class, particularly in cases of primary failure or recurrence of class-associated AEs. The efficacy of other biological agents, such as anakinra (IL1 inhibitor),^29^ abatacept (selective costimulation inhibitor),^15^ RTX (B-cell depleting antibodies)^24^ or tocilizumab (IL6 signalling inhibitor)^13^ has been confirmed in large placebo-controlled RCTs in patients with RA with inadequate response to aTNFs. However, results from existing RCTs are difficult to compare directly because they involve different patient populations, study designs and treatment strategies.^30^ In the absence of definitive RCTs comparing “head-to-head” true comparators, we examined the effect of RTX versus an alternative aTNF in a cohort study, the next best study design to answer this question.^31^ In our study, the relative benefit of RTX compared with an alternative aTNF was primarily seen in patients who had stopped their previous aTNF because of inefficacy, but not in patients who had stopped their treatment because of AEs or personal preferences. The relative benefit of RTX compared with aTNFs appeared already after a single aTNF failure and was not significantly different after more aTNF failures (fig 3; p = 0.61).

The sequence and type of aTNF switch may further affect the effectiveness of subsequent aTNF therapies. Some have highlighted greater benefits with switching between a soluble receptor (etanercept) and a monoclonal antibody (infliximab or adalimumab), compared with switches between monoclonal antibodies.^32^ Our results confirm that patients switching between an aTNF monoclonal antibody and an aTNF soluble receptor tend to have slightly larger responses (fig 2), but longitudinal improvements in DAS28 appear to be overall more favourable with RTX than with alternative aTNFs, irrespective of the type of aTNF switch (p = 0.27).

Observational studies have intrinsic limitations when analysing the therapeutic effectiveness of different agents. In particular, selection bias may occur if the assignment to RTX or an alternative aTNF is not random. In this study, the choice of the biological agent was strongly associated with preferences of individual doctors and the year of treatment initiation (median 2004 for aTNF versus 2006 for RTX) related to availability. Yet, patients receiving RTX previously had received significantly more aTNF agents that had failed, had higher disease activity levels and functional disability at baseline and were more often RF positive, suggesting that this population may have a more aggressive disease, which would tend to bias our results towards the null and result in smaller differences than in a perfectly balanced setting. We adjusted for these differences using propensity score stratification, which successfully removed observed baseline imbalances. Because baseline levels of DAS28 were higher in the RTX group, we cannot exclude the possibility that part of the effect is explained by residual regression to the mean; however, the relative benefit of RTX was also seen at lower baseline levels of DAS.

To assess the stability of our approach, we constructed alternative models using conventional multivariate regression instead of the propensity score stratification, which yielded very similar results. Qualitatively similar results were also found with propensity score adjustments and propensity score matching, suggesting the results are consistent. While we were able to control successfully the analysis for potential confounding by these variables, we cannot exclude the possibility of residual confounding or confounding by unmeasured factors. In particular, factors such as the time interval between aTNF discontinuation and new biological initiation (“wash-out period”) or the time patients were receiving their last aTNF were not consistently available for all patients and might have influenced the results if different between the two groups.

Another concern with observational studies is missing data. Our inclusion criteria permitted analysis of 66% of all patients receiving a second biological agent in the SCQM-RA cohort. Baseline disease characteristics of excluded patients were similar to those included in the analysis (data not shown), suggesting that our inclusion criteria gathered a representative sample of the population. Finally, it is difficult to establish whether the biological agents were used optimally. In this analysis, RTX was censored at the time of re-treatment, but in certain instances, patients might have benefited from earlier re-treatment. For aTNF, some patients might have been helped by higher dosages or shorter administration intervals. However, the data represent “real-world” patients and realistic clinical practice.^6^

In conclusion, these results suggest that a biological agent with a different mechanism of action, such as RTX, is an effective therapeutic alternative for patients who stopped a previous aTNF treatment owing to its ineffectiveness.

## References

[1] Lipsky PE van der Heijde DM St Clair EW Infliximab and methotrexate in the treatment of rheumatoid arthritis. Anti-Tumor Necrosis Factor Trial in Rheumatoid Arthritis with Concomitant Therapy Study Group. N Engl J Med 2000;343:1594–602 11096166 10.1056/NEJM200011303432202

[2] Weinblatt ME Kremer JM Bankhurst AD A trial of etanercept, a recombinant tumor necrosis factor receptor: Fc fusion protein, in patients with rheumatoid arthritis receiving methotrexate. N Engl J Med 1999;340:253–9 9920948 10.1056/NEJM199901283400401

[3] Weinblatt ME Keystone EC Furst DE Adalimumab, a fully human anti-tumor necrosis factor alpha monoclonal antibody, for the treatment of rheumatoid arthritis in patients taking concomitant methotrexate: the ARMADA trial. Arthritis Rheum 2003;48:35–45 12528101 10.1002/art.10697

[4] Maini R St Clair EW Breedveld F Infliximab (chimeric anti-tumour necrosis factor alpha monoclonal antibody) versus placebo in rheumatoid arthritis patients receiving concomitant methotrexate: a randomised phase III trial. ATTRACT Study Group. Lancet 1999;354:1932–9 10622295 10.1016/s0140-6736(99)05246-0

[5] Redlich K Schett G Steiner G Rheumatoid arthritis therapy after tumor necrosis factor and interleukin-1 blockade. Arthritis Rheum 2003;48:3308–19 14673982 10.1002/art.11358

[6] Finckh A Simard JF Gabay C Guerne PA Evidence for differential acquired drug resistance to anti-tumour necrosis factor agents in rheumatoid arthritis. Ann Rheum Dis 2006;65:746–52 16339288 10.1136/ard.2005.045062 PMC1798167

[7] Lutt JR Deodhar A Rheumatoid arthritis: strategies in the management of patients showing an inadequate response to TNFalpha antagonists. Drugs 2008;68:591–606 18370440 10.2165/00003495-200868050-00003

[8] Haraoui B Is there a rationale for switching from one anti-tumor necrosis factor agent to another? *J Rheumatol* 2004;31:1021–2 15170907

[9] Furst DE Gaylis N Bray V Open-label, pilot protocol of patients with rheumatoid arthritis who switch to infliximab after an incomplete response to etanercept: the opposite study. Ann Rheum Dis 2007;66:893–9 17412737 10.1136/ard.2006.068304 PMC1955098

[10] Bombardieri S Ruiz AA Fardellone P Effectiveness of adalimumab for rheumatoid arthritis in patients with a history of TNF-antagonist therapy in clinical practice. Rheumatology (Oxford) 2007;46:1191–9 17504821 10.1093/rheumatology/kem091

[11] Hyrich KL Lunt M Watson KD Outcomes after switching from one anti-tumor necrosis factor alpha agent to a second anti-tumor necrosis factor alpha agent in patients with rheumatoid arthritis: results from a large UK national cohort study. Arthritis Rheum 2007;56:13–20 17195186 10.1002/art.22331

[12] Gullick N Da Silva C Kirkham B Failure of adalimumab in patients with rheumatoid arthritis [abstract]. Arthritis Rheum 2007;56 Suppl:S183

[13] Emery P Keystone E Tony HP IL-6 receptor inhibition with tocilizumab improves treatment outcomes in patients with rheumatoid arthritis refractory to anti-tumour necrosis factor biologicals: results from a 24-week multicentre randomised placebo controlled trial. Ann Rheum Dis 2008;67:1516–23 18625622 10.1136/ard.2008.092932 PMC3811149

[14] Cohen S Emery P Greenwald M Rituximab for rheumatoid arthritis refractory to anti-tumor necrosis factor therapy: Results of a multicenter, randomized, double-blind, placebo-controlled, phase III trial evaluating primary efficacy and safety at twenty-four weeks. Arthritis Rheum 2006;54:2793–806 16947627 10.1002/art.22025

[15] Genovese MC Becker JC Schiff M Abatacept for rheumatoid arthritis refractory to tumor necrosis factor alpha inhibition. N Engl J Med 2005;353:1114–23 16162882 10.1056/NEJMoa050524

[16] Finckh A Ciurea A Brulhart L B cell depletion may be more effective than switching to an alternative anti-tumor necrosis factor agent in rheumatoid arthritis patients with inadequate response to anti-tumor necrosis factor agents. Arthritis Rheum 2007;56:1417–23 17469098 10.1002/art.22520

[17] Venkatachalam S Roskell S Suchitra R Rituximab may be more effective than switching to an alternative TNF inhibitor in rheumatoid arthritis patients who have failed other TNF inhibitors [abstract]. Rheumatology (Oxford) 2008;47 suppl:ii28

[18] Blom M Kievit W den Broeder A Comparison of the effectiveness of rituximab and a third TNF blocking agent after failure of two TNF blocking agents in daily clinical practice [abstract]. Arthritis Rheum 2008;58:S304

[19] van Vollenhoven R Carli CC The efficacy of rituximab in patients with rheumatoid arthritis (RA) who previously failed one or two anti-TNFs as compared to the efficacy of switching between anti-TNFs: a registry study [abstract]. Arthritis Rheum 2008;58:S899

[20] Uitz E Fransen J Langenegger T Clinical quality management in rheumatoid arthritis: putting theory into practice. Swiss Clinical Quality Management in rheumatoid arthritis. Rheumatology (Oxford) 2000;39:542–9 10852987 10.1093/rheumatology/39.5.542

[21] Finckh A Simard JF Duryea J The effectiveness of anti-tumor necrosis factor therapy in preventing progressive radiographic joint damage in rheumatoid arthritis: a population-based study. Arthritis Rheum 2006;54:54–9 16385495 10.1002/art.21491

[22] Prevoo ML van ’t Hof MA Kuper HH Modified disease activity scores that include twenty-eight-joint counts. Development and validation in a prospective longitudinal study of patients with rheumatoid arthritis. Arthritis Rheum 1995;38:44–8 7818570 10.1002/art.1780380107

[23] D’Agostino RB Jr Propensity score methods for bias reduction in the comparison of a treatment to a non-randomized control group. Stat Med 1998;17:2265–81 9802183 10.1002/(sici)1097-0258(19981015)17:19<2265::aid-sim918>3.0.co;2-b

[24] Cohen SB Emery P Greenwald MW Rituximab for rheumatoid arthritis refractory to anti-tumor necrosis factor therapy: results of a multicenter, randomized, double-blind, placebo-controlled, phase III trial evaluating primary efficacy and safety at twenty-four weeks. Arthritis Rheum 2006;54:2793–806 16947627 10.1002/art.22025

[25] Smolen J Kay J Doyle M Golimumab, a new human anti-TNF-alpha monoclonal antibody, subcutaneously administered every 4 weeks in patients with active rheumatoid arthritis who were previously treated with anti-TNF-alpha agent(s): results of the randomized double-blind, placebo-controlled trial [abstract]. Ann Rheum Dis 2008;**67**(suppl II):50–1

[26] Gomez-Reino JJ Carmona L Switching TNF antagonists in patients with chronic arthritis: an observational study of 488 patients over a four-year period. Arthritis Res Ther 2006;8:R29 16507128 10.1186/ar1881 PMC1526564

[27] Yazici Y Erkan D Do etanercept-naive patients with rheumatoid arthritis respond better to infliximab than patients for whom etanercept has failed? Ann Rheum Dis 2004;**63**:607–8; author reply 608. PMC1754974 15082503

[28] Karlsson JA Kristensen LE Kapetanovic MC Treatment response to a second or third TNF-inhibitor in RA: results from the South Swedish Arthritis Treatment Group Register. Rheumatology (Oxford) 2008;47:507–13 18304941 10.1093/rheumatology/ken034

[29] Buch MH Bingham SJ Seto Y Lack of response to anakinra in rheumatoid arthritis following failure of tumor necrosis factor alpha blockade. Arthritis Rheum 2004;50:725–8 15022311 10.1002/art.20115

[30] O’Dell JR Therapeutic strategies for rheumatoid arthritis. N Engl J Med 2004;350:2591–602 15201416 10.1056/NEJMra040226

[31] Boers M Understanding the window of opportunity concept in early rheumatoid arthritis. Arthritis Rheum 2003;48:1771–4 12847667 10.1002/art.11156

[32] Solau-Gervais E Laxenaire N Cortet B Lack of efficacy of a third tumour necrosis factor alpha antagonist after failure of a soluble receptor and a monoclonal antibody. Rheumatology (Oxford) 2006;45:1121–4 16510526 10.1093/rheumatology/kel054

